# Strenuous Exercise Alters Brain Creatine and Glutamate/Glutamine (Glx) in Humans: Evidence From Dynamic ^1^H‐MRS and ^1^H‐MRSI

**DOI:** 10.1096/fj.202504543R

**Published:** 2026-01-23

**Authors:** Jedd Pratt, Antonia Kaiser, Libby Henthorn, Oliver Mundell, Elise From, Aneurin J. Kennerley, Craig Sale

**Affiliations:** ^1^ Department of Sport and Exercise Sciences Manchester Metropolitan University Institute of Sport Manchester UK; ^2^ CIBM Center for Biomedical Imaging École Polytechnique Fédérale de Lausanne (EPFL) Lausanne Switzerland

**Keywords:** brain energy metabolism, exercise, magnetic resonance spectroscopy, neurometabolites, neurotransmission

## Abstract

The acute effects of exercise on brain energy metabolism are poorly understood. We used dynamic proton magnetic resonance spectroscopy (^1^H‐MRS) to quantify responses of total creatine (tCr), glutamate/glutamine (Glx), and lactate across brain regions following a single bout of strenuous exercise. Sixteen healthy adults (nine female, age: 25 ± 3 years) completed ~15 min of cycling, reaching ~85% of predicted heart rate maximum. Single‐voxel ^1^H‐MRS was acquired at 3 Tesla from frontal and visual cortices before (PRE), 15‐ (POST15), and 30‐min (POST30) following exercise. Multivoxel ^1^H‐MRSI was acquired from frontal and parietal regions PRE and 25‐min following exercise (POST25). tCr concentrations decreased by 5.9% in the frontal cortex between PRE and POST15 (6.87 vs. 6.47 mmol·L^−1^; *p* < 0.001) and remained significantly lower than PRE at POST30 (6.87 vs. 6.66 mmol·L^−1^; *p* = 0.030). Glx concentrations increased by 28.9% in the frontal cortex between PRE and POST15 (6.82 vs. 8.79 mmol·L^−1^; *p* = 0.002), returning to baseline by POST30 (*p* = 0.890). In exploratory analyses (*n* = 5 after quality control), lactate concentrations increased by 76% in the frontal cortex between PRE and POST15 (0.45 vs. 0.79 mmol·L^−1^) and remained elevated by 46% at POST30 compared to PRE (0.45 vs. 0.65 mmol·L^−1^). tCr resonance signals were 5.1% lower across the MRSI grid at POST25 compared with PRE (792.4 vs. 835.4 a.u.; *p* = 0.001), whereas Glx signals were 11.4% higher at POST25 (1349.8 vs. 1211.4 a.u.; *p* = 0.003), although region‐specific responses were shown. These findings suggest that creatine (Cr) and lactate may serve as alternative energy substrates in response to vigorous exercise, and support Glx as an indicator of excitatory neurotransmission that responds to exercise.

## Graphical abstract

Strenuous exercise induces transient, region‐specific neurochemical changes in the human brain. Dynamic proton magnetic resonance spectroscopy (^1^H‐MRS) and spectroscopic imaging (^1^H‐MRSI) revealed reductions in total creatine (tCr), and elevations in glutamate/glutamine (Glx), and lactate (Lac) in frontal and parietal brain regions. These findings suggest that creatine and lactate may serve as alternative energy substrates in response to vigorous exercise, while the changes in Glx are consistent with increased excitatory neurotransmission.

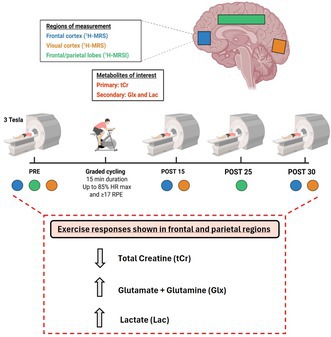

## 1 Introduction

Engaging in regular physical activity and structured exercise confer widespread benefits to human health [1, 2]. While the adaptive responses of skeletal muscle to exercise have been extensively characterized [3, 4, 5], the effects of exercise on brain are less understood. Evidence suggests that regular physical activity can protect against aging‐related cognitive decline and reduce the risk of neurological diseases, such as Alzheimer's [6, 7]. There is also evidence that exercise, particularly when performed at high intensity, may confer acute cognitive benefits [8], although the neurobiological pathways underpinning these benefits are unknown. One emerging hypothesis is that exercise favorably modulates the brain's neuroenergetic and neurochemical environment (i.e., processes relating to energy metabolism and neurotransmission), thereby supporting cognition and conferring neuroprotection [9, 10]. Difficulties in reliably assessing these changes in vivo have, however, limited our mechanistic understanding and impeded the design of targeted therapeutic strategies to support brain health.

Recent advances in magnetic resonance spectroscopy (MRS) and MR‐spectroscopic imaging (MRSI) have improved our ability to noninvasively quantify a range of neurometabolites involved in brain energy metabolism and neurotransmission [11, 12]. Although the energy requirements of the brain are invariably met by glucose at rest [13], accumulating evidence suggests that the contribution of glucose to brain energy metabolism may decrease with increasing exercise intensity [14, 15, 16]. These data indicate that other metabolites may play a central role in sustaining elevated metabolic activity during exercise and could contribute to the positive effects of exercise on brain health and cognitive function. Characterizing acute exercise responses may represent, therefore, a promising avenue for isolating neuroenergetic and neurochemical changes that may contribute to longer‐term benefits. This hypothesis is supported by evidence that acute exercise effects are predictive of changes in functional brain systems and cognition over a 12‐week aerobic training intervention [17]. Despite this, only a handful of studies have assessed MRS‐derived responses of brain metabolites to acute exercise (reviewed here: [10]).

Among the metabolites measurable via MRS, creatine (Cr) is of particular interest given its established role as a temporal and spatial energy buffer, particularly in tissues with high and fluctuating energy demands, such as brain [18, 19]. Despite its biological significance, however, Cr has been underrepresented in studies to date, with a likely contributor being the historic assumption that Cr levels are stable in the human brain and the consequent use of Cr as a reference metabolite [20]. Only two studies have assessed the effects of exercise on brain Cr; one performed at an ultrahigh magnetic field strength [7 Tesla (T)] showing significant reductions in total creatine (tCr; the pool of free Cr and phosphorylcreatine [PCr]) in the occipital cortex [21], and another performed at 3 T showing no differences in tCr in the visual cortices [22]. Notably, given that ^1^H‐MRS provides a measure of tCr, a reduction in tCr following a single bout of exercise would not be initially expected. If PCr levels were to decline in response to increased energy demand, there would be a concomitant rise in free Cr, meaning that tCr should remain stable. However, increasing evidence of dynamic alterations in tCr within skeletal muscle during exercise warrants investigation of similar mechanisms in brain. The overall paucity of evidence, combined with emerging data showing that brain Cr levels are more dynamic than previously assumed, particularly under conditions of increased metabolic demand [23, 24], provide a strong rationale for further investigation. Beyond Cr, glutamate and glutamine (combined signal referred to as Glx) are central to excitatory neurotransmission in human brain (reviewed here: [25]), and are, therefore, likely to contribute to brain's response to increased metabolic and functional demands of exercise. There is evidence that acute exercise increases brain Glx levels [22, 26], thereby indicating increased neuronal activation, although conflicting findings warrant further investigation [21, 27]. Quantifying potential changes in tCr and Glx following exercise may provide complimentary insight into neuroenergetic and neurochemical responses that may enable us to better understand the beneficial effects of exercise on brain health.

The a priori aim of the present study was to examine the effects of a single bout of strenuous exercise on tCr levels in the human brain. Following data collection, we also made an a posteriori decision to examine the effects of exercise on brain Glx levels. Dynamic ^1^H‐MRS and ^1^H‐MRSI were used to capture temporal and spatial responses across multiple brain regions. Based on known physiological mechanisms and existing literature, we hypothesized that brain tCr levels would remain unchanged following exercise, whereas brain Glx levels would increase reflecting enhanced neurotransmission.

## 2 Methods

### 2.1 Study Sample and Design

Sixteen healthy young adults (nine female) aged 20–30 years were recruited for this study. All participants were recreationally active, defined as engaging in at least 45 min of moderate‐to‐vigorous physical activity on three or more days per week. All participants completed a magnetic resonance safety form and physical activity readiness questionnaire to confirm suitability for having an MR scan and performing high‐intensity exercise. Inclusion criteria were: aged 18–35 years; free from musculoskeletal, cardiovascular, or neurological contraindications to strenuous exercise; no contraindication to MR scanning; no neurological disorder affecting typical brain metabolism; no habitual Cr supplementation within the previous 3 months; no use of medications affecting cardiovascular function and responses to exercise; and no caffeine consumption within 6 h of testing. Full ethical approval was granted by Manchester Metropolitan University's research ethics committee (ref: 62998), and all participants provided written informed consent.

**FIGURE 1:**
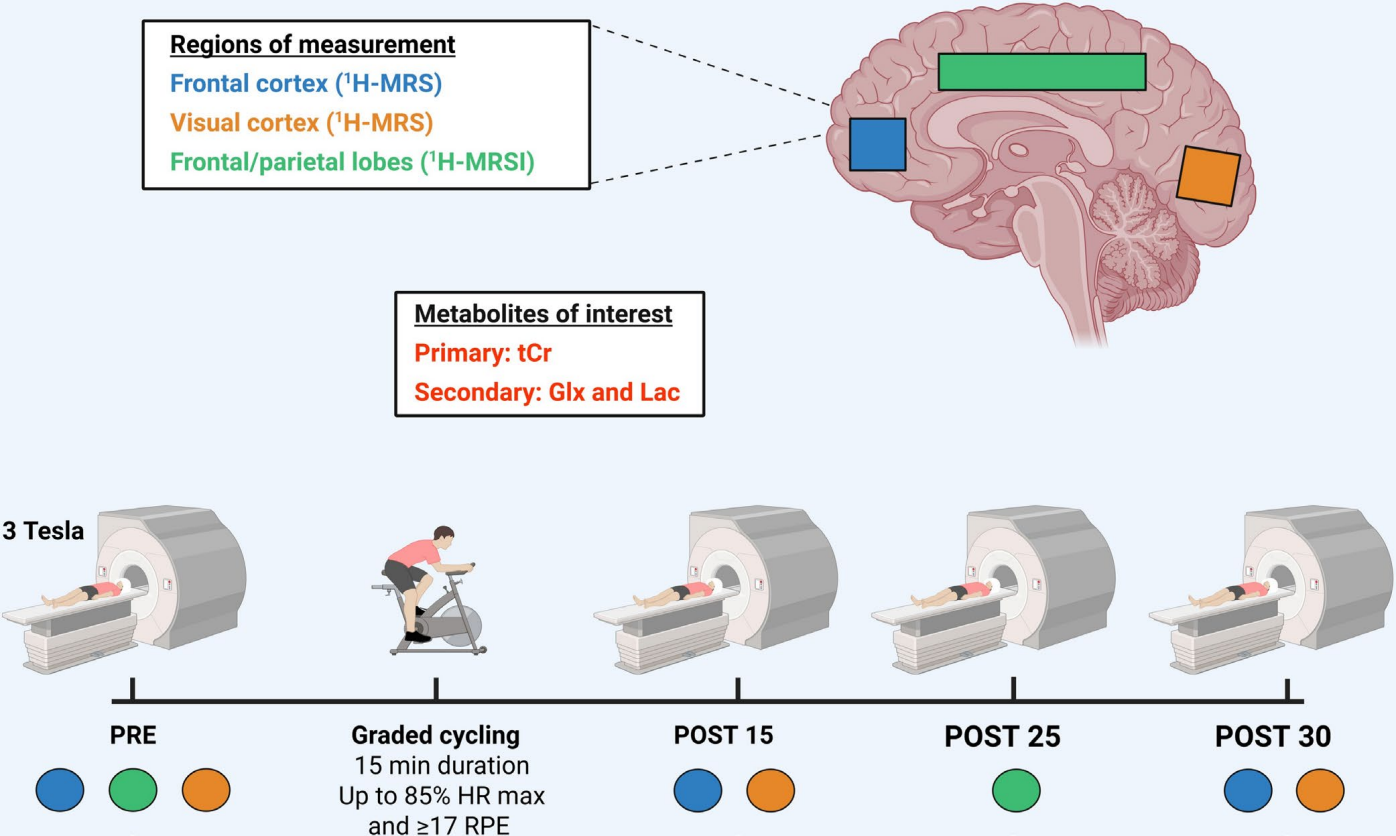
Schematic overview of study design. Color coding corresponds to brain regions assessed at each timepoint and timings shown are minutes postexercise cessation. ^1^H‐MRS, proton magnetic resonance spectroscopy; Glx, combined glutamate and glutamine signal; HR, heart rate; Lac, lactate; RPE, rate of perceived exertion; tCr, total creatine.

A schematic of the study design can be seen in Figure 1. Each participant had 25 min of baseline MR measurements taken (PRE) including single‐voxel proton magnetic resonance spectroscopy (^1^H‐MRS) in the frontal and visual cortices, and multivoxel 2D magnetic resonance spectroscopic imaging (^1^H‐MRSI) through an axial slab positioned above the corpus callosum (full details of MR acquisitions and processing are detailed below). Participants were then removed from the MR scanner and underwent a graded, high‐intensity exercise test in an adjacent laboratory. The test was performed on an Excalibur Sport cycle ergometer (Lode, Groningen, The Netherlands), using a RAMP protocol wherein the resistance started between 60 and 90 W (females and males) and increased by approximately 30 W every 3 min until the participant reached ≥ 85% of predicted HR maximum (220‐age) and at least 17 (i.e., very hard) on the borg rate of perceived exertion (RPE) scale. Heart rate was recorded continuously throughout the test using a Polar heart rate monitor and chest strap, and RPE were recorded in the last 30 s of every stage. The exercise protocol was selected to be comparable to existing literature on MRS‐derived changes in brain metabolites following exercise [21, 26]. Upon completion of the exercise test, participants were repositioned (within 2–3 min) in the MR scanner for post assessments, including repeated single voxel ^1^H‐MRS taken 15 min (POST 15) and 30 min (POST 30) postexercise cessation, and ^1^H‐MRSI taken at 25 min postexercise cessation (POST 25) (all time‐points were ±1 min). The overall duration of the testing visit was 90 min.

### 2.2 Magnetic Resonance

**FIGURE 2:**
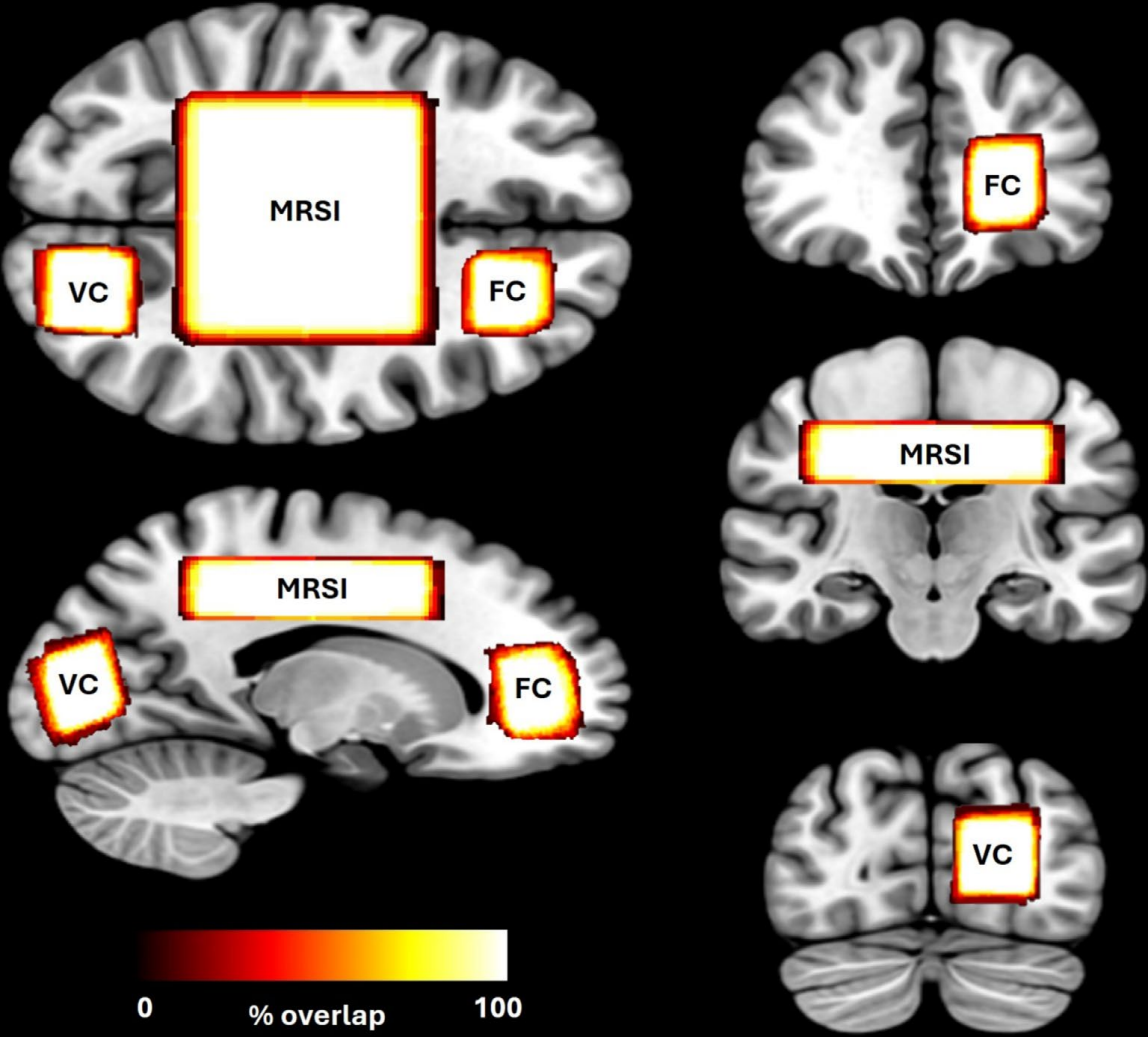
Average overlap between repeated voxel localization for each region. FC, frontal cortex; MRSI, magnetic resonance spectroscopic imaging; VC, visual cortex.

MR imaging and spectroscopy were performed on a 3 T Siemens MAGNETOM Vida (Siemens Healthineers, Erlangen, Germany), running XA6 software and using a 20‐channel receive‐only head coil. Care was taken when positioning each participant on the scanner to ensure their comfort and maximize compliance with stillness during the acquisitions. Several pieces of cushioning were positioned around the participant's head and under the participant's legs to dampen head movements and mitigate any lumbar pressure during the acquisitions. Two localizers integrating distortion correction and a high‐resolution *T*_1_‐weighted magnetization prepared rapid gradient echo (MPRAGE) sequence acquired in the sagittal plane ([repetition time] TR = 2100 ms; [echo time] TE = 2.58 ms; flip angle = 8°; slice thickness 1 mm; slice number = 176; [field of view] FoV = 250 mm; voxel size = 1 × 1 × 1 mm) enabled accurate voxel placement as previously demonstrated [28]. Sagittal images were converted into axial and coronal planes, and all three planes were used as references to position the VOI in each brain region. Anatomical landmarks and the previous measurement (for postmeasurements) were used to enhance relocalization accuracy (Figure 2).

^1^H‐MRS was acquired from a 20 × 20 × 20 mm voxel placed in the frontal cortex and visual cortex of the left hemisphere using a point resolved spectroscopy (PRESS) sequence ([repetition time] *T*_*R*_ = 2000 ms; [echo time] *T*_*E*_ = 30 ms; 130 averages; bandwidth = 1200 Hz; spectral points = 1024). Siemens brain automated static magnetic field (*B*_0_) shimming and standard chemical shift selective (CHESS) suppression with 50 Hz bandwidth were used. Immediately after the water‐suppressed acquisition, using the same parameters, 10 water‐unsuppressed averages were taken, which were later used for eddy‐current correction and absolute metabolite quantification. Multivoxel 2D ^1^H‐MRSI was acquired from a 160 mm FOV (16 × 10 × 15 mm voxels, 15 mm slice thickness) placed above the corpus callosum using a localizer and composite localization with adiabatic slice selective excitation and refocusing (cLASER) sequence (TR = 1700 ms; TE = 40 ms; 3 averages; bandwidth = 1200 Hz). Data were oversampled during acquisition and subsequently reconstructed to an effective 80 mm FOV (8 × 10 × 15 mm voxels) for analyses. All details for the MRS/I acquisition parameters can be found in the MRSinMRS Checklist presented in Table S1 [29].

### 2.3 Data Processing and Quality Assurance

^1^H‐MRS data were processed in OSPREY (v.2.6.0), using water‐unsuppressed signals as references to determine absolute tCr concentrations. OSPREY is a state‐of‐the‐art MATLAB‐based toolbox that enables processing, analysis, and quantification of metabolite concentrations in vivo [30]. Standard preprocessing steps included spectral registration, eddy current correction, zero‐ and first‐order phase corrections, baseline corrections and lipid filtering, and data were quantified using the in‐built LCModel fitting algorithm (Table S1). Default ^1^H‐MRS basis sets based upon Siemens PRESS were used for metabolite quantification, and included: alanine, aspartate, Cr, gamma‐aminobutyric acid, glutamine, glutamate, glycerophosphorylchlorine, guanidinoacetate, myoinositol, lactate, *N*‐acetylaspartate, *N*‐acetylaspartylglutamate, phosphorylcholine, PCr, scylloinositol, and taurine. Due to the absence of multivoxel analyses capabilities within OSPREY, ^1^H‐MRSI data were analyzed using TARQUIN (v.4.3.10) an established automated analysis tool, comparable to LCModel, that allows for the processing and analysis of multivoxel ^1^H‐MRSI data [31]. We have previously shown both OSPREY and TARQUIN to be highly precise in repeatedly determining MRS data, although significant differences were shown in absolute metabolite concentrations determined across packages [28]. As such, to avoid potential quantification conflicts across OSPREY and TARQUIN, we opted not to calculate absolute concentrations for the ^1^H‐MRSI data and instead expressed metabolites in relation to signal intensity and fold change across the repeated measurements. Following the consensus guidelines for reporting standards in MRS [29], quality metrics, including full width at half‐maximum (FWHM), signal‐to‐noise ratio (SNR), and Cramér–Rao lower bounds (CRLB) of the metabolites of interest were determined during the model‐fitting procedure for each spectrum (Table S2). Absolute metabolite concentrations were adjusted for water‐content in different tissue fractions using SPM12 segmentation within OSPREY. The overlap of voxel placement between repeated measurements was calculated for each brain region to establish the consistency of measurement location (Figure 2).

### 2.4 Metabolites of Interest

The a priori metabolite of interest was Cr. As free Cr and PCr both resonate at 3.03 ppm (CH_3_—methyl group) and 3.92 ppm (CH_2_—methylene group), it is not possible to distinguish their peaks at 3 T. Therefore, the sum of the two peaks was considered as the primary tCr value, while supplementary analyses of the tCr Cr‐CH_2_ and tCr Cr‐CH_3_ peaks were also performed. An a posteriori decision was made to also assess glutamate and glutamine. Due to the challenges associated with delineating the glutamate and glutamine signals at 3 T, their combined signal, hereafter referred to as Glx, was considered.

Similarly given the role of lactate in brain energy metabolism during exercise [32, 33], we also made an a posteriori decision to perform exploratory analyses of brain lactate responses to exercise where significant changes in tCr and Glx were shown. As the MRS sequences were not optimized for lactate determination, a broader threshold of model fitting (CRLB < 40%) was considered appropriate. Given the exploratory nature of these data, no statistical analyses were performed, and significant attention was given to visual inspection of the doublets at 1.32 ppm.

### 2.5 Statistical Analyses

All data are presented as mean ±1 standard deviation (SD), unless stated otherwise. Normality of data was confirmed by Shapiro–Wilk tests. One‐way repeated measures ANOVAs were used to assess changes in tCr and Glx and spectral quality metrics following exercise. Where a significant main effect of time was shown, Tukey's honest significant difference post hoc tests were used to isolate specific differences between time points (i.e., PRE vs. POST 15 vs. POST 30). Greenhouse–Geisser corrections were applied to account for sphericity violations. Student's paired *t*‐tests were used to determine changes in tCr and Glx across the ^1^H‐MRSI grid before and after exercise (i.e., PRE vs. POST 25). Pearson correlation coefficients were used to determine the relationship between change in tCr FWHM and change in tCr and Glx concentrations. Linear mixed models were used to determine whether changes in tCr concentrations following exercise were independent of concurrent changes in tCr FWHM (time: fixed effect; tCr FWHM: continuous covariate; subjects: random intercept) [34]. The threshold for statistical significance was *p* < 0.05 for all tests. Statistical analyses were performed using SPSS (v.31.0; IBM SPSS Inc., Chicago, IL, USA), and data visualizations were developed using GraphPad Prism (v.9.3.1; GraphPad, San Diego, CA, USA) and MATLAB (vR2023b; MathWorks Inc., Natick, MA, USA).

## 3 Results

### 3.1 Participant Characteristics and Exercise Intensity

The mean age, height, and body mass of the participants was 25.2 ± 3.2 years, 1.71 ± 0.10 m, and 64.6 ± 11.9 kg, respectively. Participants exercised, on average, to 87% ± 2% of their maximum HR over an average test length of 15 ± 2 min. The average HR during the exercise testing was 142 ± 4 beats per minute. The average RPE in the last stage of the test was 18 ± 1 and the average maximum resistance was 168 ± 28 W (females: 149 ± 12; males: 193 ± 24).

### 3.2 Spectral Quality and Voxel Relocalization Accuracy

All spectral data met the following minimum quality thresholds for inclusion in analyses (CRLB < 20% [< 40% allowed for lactate], SNR > 10 and FWHM < 15 Hz). Table S2 displays spectral quality metrics, including SNR, FWHM, and CRLB, for each brain region across each time point, while Figure S1 presents representative raw spectra from each brain region. The average CRLBs for tCr and Glx ranged between 3% and 6% in the frontal cortex, 2% and 4% in the visual cortex, and 2% and 7% across the MRSI grid, indicating a highly reliable model fit. The percentage overlap between repeated voxel relocalizations for each brain region can be seen in Figure 2. The average voxel overlap between repeated measures was ≥ 90% for all brain regions.

### 3.3 Responses of Brain tCr and Glx to Exercise

**FIGURE 3:**
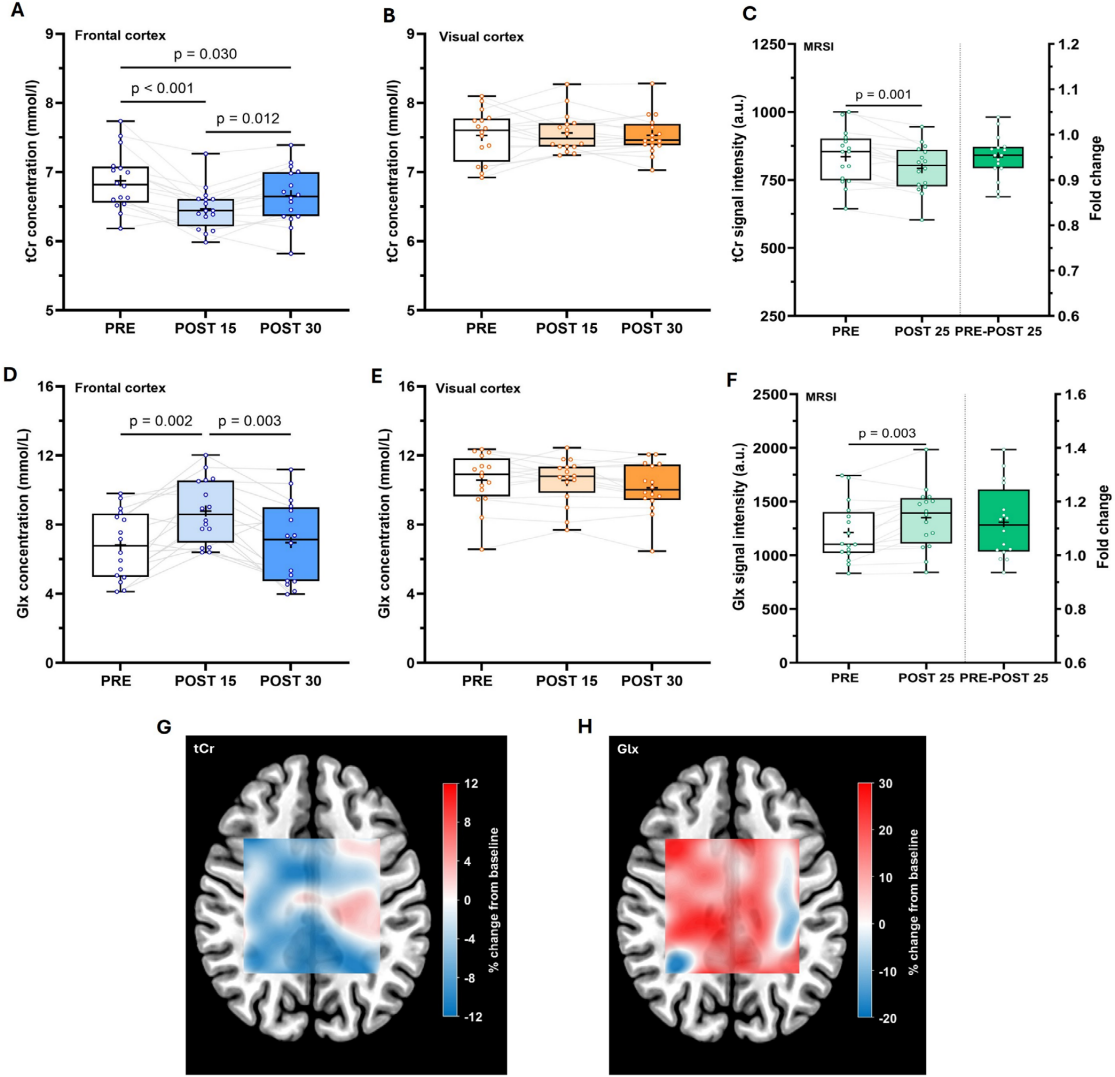
Response of total creatine (tCr) and glutamate/glutamine (Glx) following exercise in the frontal cortex (A + D), visual cortex (B + E), and MRSI grid (C + F: Averaged across grid; G + H: Regional responses).

Significant main effects of time were shown for tCr (*F*_(1.8, 26.3)_ = 16.3; *p* < 0.001; $\eta_{p}^{2}$ = 0.52) and Glx concentrations (*F*_(1.6, 23.8)_ = 6.5; *p* = 0.009; $\eta_{p}^{2}$ = 0.42) in the frontal cortex, but not in the visual cortex (all *p* > 0.05). Post hoc testing revealed that tCr concentrations decreased by 5.9% in the frontal cortex between PRE and POST 15 (6.87 vs. 6.47 mmol·L^−1^; *p* < 0.001) and remained significantly lower (by 3.2%) than PRE levels at POST 30 (6.87 vs. 6.66 mmol·L^−1^; *p* = 0.030), although signs of a gradual return to baseline concentrations were present (Figure 3). In contrast, Glx concentrations increased by 28.9% in the frontal cortex between PRE and POST 15 (6.82 vs. 8.79 mmol·L^−1^; *p* = 0.002) but were not significantly different from PRE levels at POST 30 (6.82 vs. 7.09 mmol·L^−1^; *p* = 0.890) (Figure 3).

Multivoxel ^1^H‐MRSI revealed that, overall, tCr resonance signals were significantly lower (by 5.1%) across the grid at POST 25 compared with PRE levels (792.4 vs. 835.4 a.u.; *t*_(15)_ = 4.0; *p* = 0.001; $\eta_{p}^{2}$ = 0.52), whereas Glx resonance signals were significantly higher (by 11.4%) at POST 25 compared with PRE levels (1349.8 vs. 1211.4 a.u.; *t*_(15)_ = 3.6; *p* = 0.003; $\eta_{p}^{2}$ = 0.47). Although global decreases and increases were shown in tCr and Glx across the MRSI grid, region‐specific responses were shown (Figure 3). Notably, in the areas where the left primary motor cortex (M1) and somatosensory cortex (S1) were captured within the MRSI grid, opposite effects were shown for tCr and Glx following exercise (i.e., tCr increased and Glx decreased) (Figure S2 shows MRSI heatmap with overlaid M1 and S1 regions).

### 3.4 Additional Analyses of tCr Responses in the Frontal Cortex

Significant main effects of time were shown for tCr/tNAA concentrations (*F*_(1.8, 26.7)_ = 15.5; *p* < 0.001; $\eta_{p}^{2}$ = 0.51) and tCr/tNAA peak height (*F*_(2.0, 29.4)_ = 9.5; *p* < 0.001; $\eta_{p}^{2}$ = 0.39). Posthoc testing showed that tCr/tNAA concentrations and tCr/tNAA peak height decreased by 7.7% and 9.3% in the frontal cortex between PRE and POST 15 (0.62 vs. 0.57; *p* < 0.001; 0.60 vs. 0.54; *p* = 0.002) and remained significantly lower (by 4.8% and 6.2%) than PRE levels at POST 30 (0.62 vs. 0.59; *p* = 0.003; 0.60 vs. 0.56; *p* = 0.043). When analyzed according to tCr resonance peaks (i.e., Cr‐CH_2_ and Cr‐CH_3_), reductions in Cr‐CH_2_ tCr concentrations contributed to the majority (87%) of the change in the overall tCr concentration between PRE and POST 15 (1.71 vs. 1.35 mmol·L^−1^; *p* = 0.025).

tCr SNR and CRLB remained comparable at all timepoints (SNR: 74.7 vs. 71.9 vs. 72.0; CRLB: 2.6 vs. 2.6 vs. 2.8 for PRE, POST 15, and POST 30; all *p* > 0.05). A main effect of time was shown for tCr FWHM (*F*_(1.6, 24)_ = 4.7; *p* = 0.025; $\eta_{p}^{2}$ = 0.24), which was driven by a slight broadening of the tCr FWHM was between PRE and POST 15 (6.6 vs. 8.0 Hz; *p* = 0.034), despite the water FWHM remaining comparable at all timepoints (8.7 vs. 8.4 vs. 9.0 Hz; *p* > 0.05). Change in tCr FWHM was weakly correlated to change in tCr and Glx concentrations (*r* = 0.31 and 0.20; *η* ^2^ = 0.10 and 0.04; both *p* > 0.05). The main effect of time on tCr concentrations remained significant when change in tCr FWHM was considered as part of a linear mixed model (*F*_(2, 44)_ = 3.5; *p* = 0.026; $\eta_{p}^{2}$ = 0.17), with significant differences shown between PRE and POST 15 (6.96 vs. 6.60 mmol·L^−1^; *p* = 0.012), but not between PRE and POST 30 (6.96 vs. 6.76 mmol·L^−1^; *p* = 0.164).

### 3.5 Response of Brain Lactate to Exercise

**FIGURE 4:**
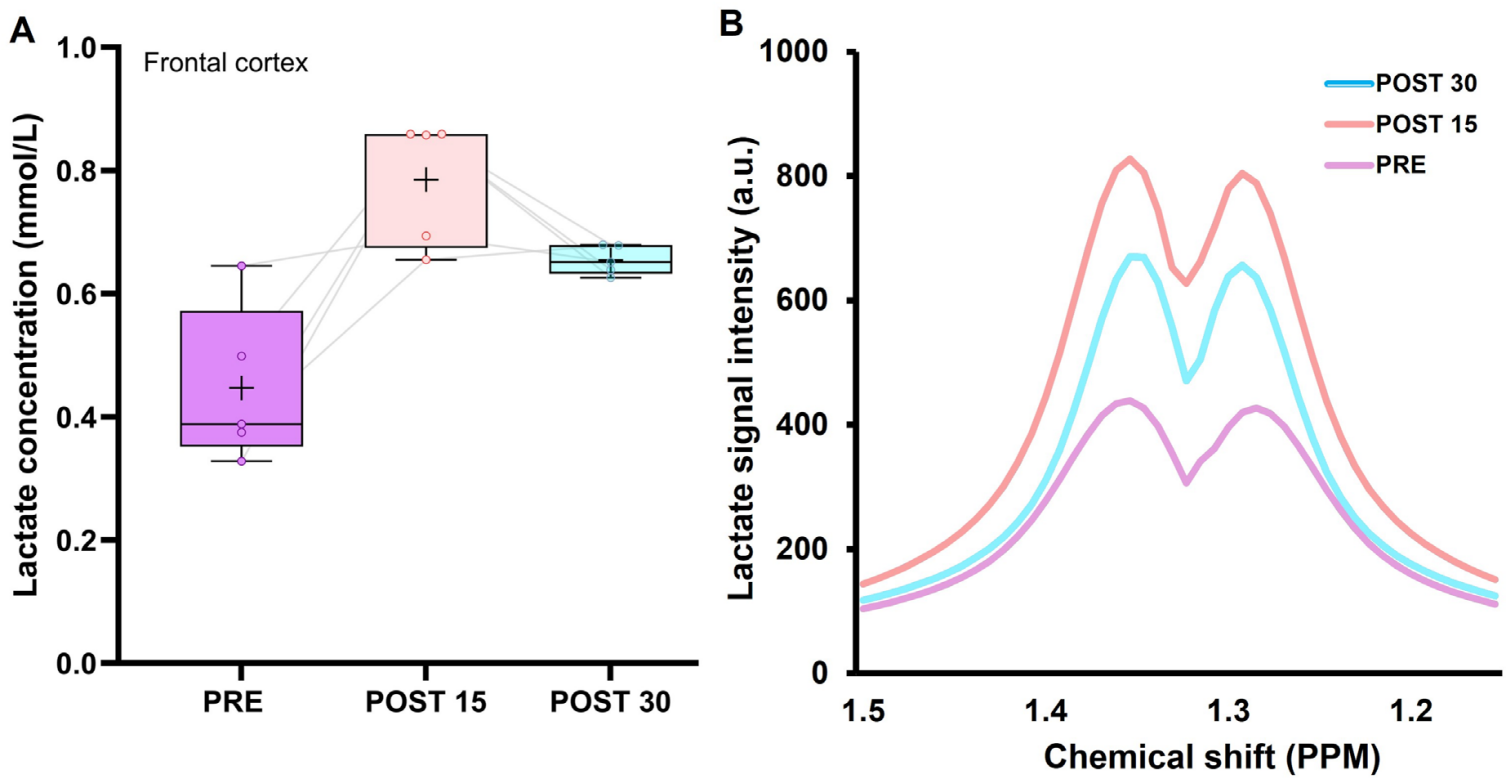
Response of brain lactate concentrations to exercise (A) and representative lactate doublets before and after exercise (B).

Lactate concentrations (*n* = 5 after thorough quality control) increased by 76% in the frontal cortex between PRE and POST 15 (0.45 vs. 0.79 mmol·L^−1^) and remained elevated (by 46%) at POST30 compared to PRE levels (0.45 vs. 0.65 mmol·L^−1^) (Figure 4). These data were supported by manual inspection of the lactate doublets centered around 1.32 ppm, which showed visibly higher peaks following exercise.

## 4 Discussion

This is the first study to combine dynamic ^1^H‐MRS and ^1^H‐MRSI to assess the acute effects of exercise on neurometabolite levels in multiple regions of the human brain. We show that a single bout of strenuous exercise induces region‐specific alterations in tCr and Glx in regions involved in motor control, executive functioning, and emotion regulation. These data suggest that acute neurochemical and neuroenergetic responses may underpin the brain's adaptive capacity to exercise, and that energy substrates other than glucose may be increasingly relied upon to maintain brain function in response to periods of high‐intensity activity.

### 4.1 tCr Responses

In contrast to expectation, vigorous exercise led to acute decreases of tCr levels in the frontal cortex and in regions of the MRSI grid covering the parietal lobe. Interestingly, such findings align with evidence from skeletal muscle, where similar reductions in ^1^H‐MRS derived tCr concentrations have been shown following fatiguing exercise [35, 36, 37]. Given that tCr reflects the combined pool of free Cr and PCr, this reduction is not easily attributable to PCr utilization, although a decline in PCr is physiologically likely. The challenge in interpretation lies in the fact that if PCr levels were depleted to meet the increased metabolic demands of exercise, a proportional increase in free Cr would be expected, and thus, the overall pool of tCr would remain stable. As such, the observed reduction in tCr may reflect a more complex physiological, or perhaps biophysical, response. Although direct assessment of the mechanistic pathways was beyond the scope of the present study, we propose, herein, several hypotheses that may help contextualize our findings.

**FIGURE 5:**
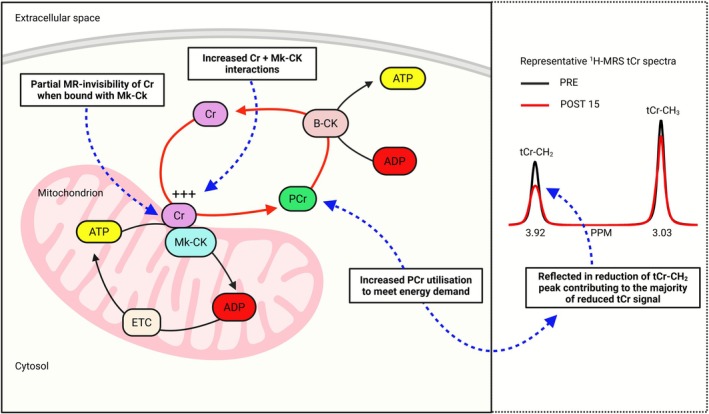
Potential mechanisms underpinning the observed reduction in brain tCr following strenuous exercise. ^1^H‐MRS, proton MR‐spectroscopy; ADP, adenosine diphosphate; ATP, adenosine triphosphate; B‐CK, brain creatine kinase; CH_2_, methylene group; CH_3_, methyl group; Cr, free creatine; ETC, electron transport chain; Mk‐CK, mitochondrial creatine kinase; MR, magnetic resonance; PCr, phosphorylcreatine; POST 15, 15 min following exercise cessation; PPM, parts per million; PRE, before exercise; tCr, total creatine.

The first and perhaps most plausible explanation is that intrinsic differences in spectral and relaxation properties between Cr and PCr become more pronounced as their relative concentrations shift in response to the energetic demands of strenuous exercise [24, 35, 36, 37]. In this scenario, changes to the dynamic PCr ↔ Cr + Pi equilibrium favor the formation of Cr, which may become less visible due to its distinct molecular signatures [35, 36, 37, 38]. For example, Cr and PCr have different relaxation times and Cr has a considerably faster proton exchange rate, which can contribute to signal attenuation, particularly in cases of increased metabolic demand where Cr interacts transiently with Cr kinase [39]. These factors may cause partial signal loss that disproportionately affects Cr, creating the appearance of a reduced tCr signal on ^1^H‐MRS, despite tCr content remaining stable [24, 39, 40, 41]. Importantly, this phenomenon appears to be specific to the Cr‐CH_2_ (methylene) resonance, where PCr is thought to contribute more significantly to the tCr signal, than at the Cr‐CH_3_ (methyl) resonance [35, 36, 37]. It is possible that the Cr‐CH_2_ resonance is more closely linked with PCr than the Cr‐CH_3_ resonance, as the CH_2_ protons are more sensitive to changes in the local chemical environment and molecular interactions associated with the phosphate group in PCr. This is supported by previous ^1^H‐MRS studies in skeletal muscle, which showed similar decreases in tCr following exercise, and that these changes were consistently driven by reductions in the Cr‐CH_2_ resonance [35, 36, 37]. Particularly noteworthy, is that in two of these studies, the reduction of the Cr‐CH_2_ peak closely scaled with reductions in PCr as determined using ^31^P‐MRS [35, 37]. The present study extends this effect to brain, showing that changes in brain tCr were largely accounted for by reductions in the Cr‐CH_2_ signal. Under this interpretation, decreases in ^1^H‐MRS‐detectable brain tCr following strenuous exercise may be linked to PCr depletion and partial MR invisibility of Cr, that are likely initiated during exercise and which persist into the early recovery period, before energy homeostasis is regained (Figure 5). Further studies employing ^31^P‐MRS are, however, needed to confirm this mechanism.

Another possibility is that exercise induces a temporary redistribution of Cr into MR‐invisible intracellular compartments, such as through movement between cytosolic and mitochondrial pools as part of the PCr‐Cr conversion cycle [18, 19]. This redistribution may involve Cr binding to Cr kinase, macromolecules, or translocation into mitochondrial compartments, thereby altering the local magnetic environment and reducing MR visibility [40, 42, 43]. This hypothesis is supported by the slight broadening of the tCr peak following exercise, despite the water linewidth remaining stable. This phenomenon, also shown in skeletal muscle [35, 36], suggests that exercise increases heterogeneity in the magnetic environment of the tCr pool. Although speculative, this could be driven by increased compartmentalization or subcellular translocation which could expose a portion of the tCr pool to environments with altered *T*_2_ relaxation times or increased magnetic susceptibility variations, thereby increasing linewidth and reducing MR signal intensity [38, 44, 45]. Magnetization transfer studies have also suggested that a portion of the tCr pool in brain may exist in a partially bound, MR‐invisible state, likely due to increased binding to Cr kinase, which may become more pronounced in response to vigorous exercise where there is increased energy demand (Figure 5) [41, 42, 46].

A further consideration is that strenuous exercise induces changes in cerebral blood flow and oxygenation which can influence tissue relaxation properties and thereby affect MR signal intensity [47, 48]. Increased perfusion and oxygen delivery may shorten *T*_1_ relaxation times, while local changes in deoxyhemoglobin concentration can alter *T*_2_ and *T*_2_* relaxation [49, 50, 51]. These effects could disproportionately impact metabolites with shorter *T*_2_ values, such as Cr, leading to partial signal attenuation independent of actual concentration changes. However, given that the water FWHM remained comparable at all time points, suggesting minimal global susceptibility changes [48], their contribution to the region‐specific responses in tCr shown in the present study is likely to be limited. Nevertheless, future studies incorporating quantitative *T*_1_/*T*_2_ mapping or BOLD imaging alongside MRS will be essential for distinguishing relaxation‐driven effects from true metabolite changes.

The alterations shown in tCr should also be considered within the context of postexercise recovery and the restoration of brain energy homeostasis. Following strenuous exercise, the PCr‐Cr system plays a central role in buffering ATP resynthesis and re‐establishing resting energy stores, a process that persists after cessation of exercise [37, 52]. In this context, the reductions shown in ^1^H‐MRS‐detectable tCr may reflect an intermediate recovery state in which energetic homeostasis has not yet been fully restored. This is supported by prior arteriovenous difference studies indicating that cerebral metabolic ratios remain altered into the postexercise recovery period (up to ~30 min) [53, 54], consistent with ongoing metabolic changes following exercise. Therefore, the present findings likely reflect neurometabolic processes associated with early postexercise recovery and the involvement of the PCr‐Cr system in regaining brain energy homeostasis. Although these are plausible hypotheses that may help contextualize our data, future mechanistic studies are needed to clarify the extent of their contribution. Regardless of pathway, however, our findings extend previous work in skeletal muscle, showing that strenuous exercise also induces an acute, postexercise decrease in the MR‐visible pool of tCr in certain regions of human brain.

### 4.2 Glx Responses

The present study strengthens existing literature showing that Glx (glutamate + glutamine) levels are transiently increased following acute exercise in several brain regions. Glutamate is the primary excitatory neurotransmitter in the human brain and is a well‐established mediator of memory consolidation and learning, particularly through long‐term potentiation which strengthens synaptic connections and supports cognitive function [55, 56, 57]. The combined Glx signal, as measured by ^1^H‐MRS is reflective of glutamatergic metabolism, whereby synaptically released glutamate is taken up by astrocytes, converted into glutamine, and transported back to neurons where it can be converted back to glutamate for reuse in neurotransmission [58]. This glutamate‐to‐glutamine conversion is critical for neuroprotection as excessive accumulation of glutamate can be excitotoxic and lead to neuronal apoptosis [59].

Although conflicting findings have been shown [21, 27], the literature broadly suggests that exercise may acutely modulate Glx levels in a way that supports neuroplasticity and neurotransmission. Our findings align with previous reports demonstrating that Glx levels in human brain are significantly increased following a single bout of vigorous exercise, compared with a resting state [22, 26, 60]. The transient increase in Glx in response to strenuous exercise may reflect an acute enhancement to neurotransmitter cycling and overall neuronal activation, that could underpin some of the immediate neurophysiological benefits of exercise. When repeated over time, such effects may contribute to longer‐term brain adaptations associated with regular physical activity. Consistent with this hypothesis, previous work has shown that repeated exposure to high‐intensity interval training increases brain Glx concentrations, and that these changes correlate with improvements in working memory and cardiorespiratory fitness among adolescents [61]. There is a need, nevertheless, for future studies to determine the extent to which transient changes in Glx following exercise are associated with acute functional improvements and whether these responses, when accumulated over time, confer additional benefits to brain health and cognitive function.

### 4.3 Lactate Responses

In a small subset of participants where lactate determination was possible, lactate levels increased substantially in the frontal cortex following strenuous exercise. We chose to focus upon the frontal cortex given the concurrent decreases in tCr concentrations in this region and the corresponding likelihood that it was particularly metabolically active in response to exercise. Although exploratory, these data align with previous studies showing that strenuous exercise transiently increases brain lactate concentrations [21, 26], which may be due to a combination of local production and increased peripheral uptake [62, 63]. Given that brain glucose uptake and metabolism are suggested to decrease with increasing exercise intensity [14, 15, 16], the elevations in brain lactate shown in the present study may reflect a compensatory mechanism to support ATP production and the restoration of brain energy homeostasis during early recovery. In this context, lactate may serve as an alternative energy substrate, helping to restore energy balance following exercise in the presence of altered substrate utilization and increased metabolic demands [32, 33]. Additionally, the elevations shown in lactate following exercise may reflect the role of lactate utilization in restoring brain energy metabolism following periods of high‐intensity exercise. While these results should be interpreted considering the limited sample size and suboptimal MR‐sequences for lactate detection, they support the growing recognition that lactate may be central to the brain's adaptive response to metabolic stress.

### 4.4 Relevance of Brain Region

An interesting point of discussion is the localization of responses in tCr and Glx, which were primarily shown within the frontal cortex and parts of the parietal lobe. We did not observe any effect of exercise in the visual cortex, suggesting that exercise induces responses that predominantly involve regions associated with motor control, executive function, and emotional processing. This is consistent with evidence from acute exercise interventions, which most commonly report improvements across domains of cognitive function that are dependent upon the frontal cortex [8]. Our findings strengthen these data showing that neuroenergetic and neurochemical processes in the frontal cortex may also be particularly responsive to exercise.

Notably, areas of the left primary motor cortex and somatosensory cortex captured within the MRSI grid showed opposite patterns to the frontal cortex (i.e., increases in tCr and decreases in Glx). These findings align with previous reports that motor and frontal regions exhibit contrasting responses in glucose metabolism during high‐intensity exercise; specifically, the motor cortex shows increases whereas frontal regions and global measures show decreases [14]. It is therefore reasonable to speculate that region‐specific differences in glucose metabolism may have contributed to the variability in tCr and Glx responses, as glucose availability in some areas may have more effectively met local energy demands. Collectively, these findings highlight the region‐specific and complex nature of neuroenergetic and neurochemical responses to exercise in brain. Future research in this field should integrate an assessment of multiple brain regions to better understand the spatial variability in brain energy metabolism and neurotransmission surrounding exercise, and how this may relate to the functional benefits associated with regular exercise.

### 4.5 Limitations

There are several limitations to acknowledge. Firstly, we did not employ ^31^P‐MRS and, as such, the response of brain PCr to exercise remains to be determined. Secondly, measurements were taken immediately following, but not during exercise, and, as such, the extent to which the responses shown reflect those occurring during exercise remains to be determined. Directly assessing MR‐derived responses during exercise is challenging, however, as exercise modalities within an MR‐scanner are not fully representative of exercise performed outside the scanner and may induce different metabolic responses. Thirdly, due to practical constraints, the final MRS measurement was taken 30 min following exercise cessation, limiting our ability to fully capture the recovery timeline of metabolite changes to baseline. Fourthly, the MR sequences were optimized for the assessment of tCr, which was the primary focus of the study; however, this may have led to the quantification of other metabolites, particularly lactate, being suboptimal. Reliable lactate detection in the human brain typically requires longer echo times than those used in the present study [11], which likely accounts for the absence of detectable lactate signals in several participants. Nevertheless, significant care was given during all data collection, processing, and analysis steps to ensure high‐quality output data, as reflected in all spectra and model fit quality metrics.

## 5 Conclusion

A single bout of strenuous exercise induces transient, region‐specific alterations in tCr, Glx, and lactate levels in young adult brain. These findings add to the emerging body of evidence that alternative energy substrates to glucose are increasingly relied upon to maintain brain function in response to metabolically demanding conditions. Longitudinal studies are warranted to determine the extent to which these acute neuroenergetic and neurochemical responses following exercise contribute to the improvements in brain health and cognitive function associated with regular physical activity.

## Author Contributions

Jedd Pratt: conceptualization, data curation, formal analysis, investigation, methodology, visualization, writing – original draft, writing – review and editing; Antonia Kaiser: investigation, methodology, writing – review and editing; Libby Henthorn: investigation, writing – review and editing; Oliver Mundell: investigation, writing – review and editing; Elise From: investigation, writing – review and editing; Aneurin J. Kennerley: investigation, formal analysis, methodology, writing – review and editing; Craig Sale: conceptualization, investigation, methodology, writing – review and editing.

## Funding

The authors have nothing to report.

## Conflicts of Interest

C.S. has current research funding from Myositis UK, Balchem Inc., Iovate Health Sciences Inc., and Vision is Sport Foundation (for ADIDAS). C.S. has previously received research funding from UK Ministry of Defense, Birmingham City University, Fundação de Amparo à Pesquisa do Estado de São Paulo, Natural Alternatives Inc., Irish Research Council, Enterprise Partnership Scheme, Coventry University, English Institute of Sport, Ciência sem Fronteiras, GlaxoSmithKline High Performance Laboratory, NOW Foods Ltd., Santander Research Mobility Grant, NHS Nottingham City, Deltex Medical, EAS Sports Nutrition, and Cytodine Technologies. C.S. is also a paid member of the Myprotein Performance Advisory Board and is Editor‐in‐Chief of The Journal of Nutritional Physiology, for which he receives an honorarium from The Physiological Society. C.S. has in the past received payment/honoraria/support in kind (predominantly for the production and delivery of lecture material) from Natural Alternatives Inc., PepsiCo (GSSI), GlaxoSmithKline, English Institute of Sport, Dairy Council, Dairy Council Norther Ireland, FSI Nutrition, Guru Performance Ltd., Institute of Performance Nutrition, Sportsoracle, WE Nutrition, ISSN, Nutrition X, and Leaders in Performance. C.S. has attempted to list all potentially relevant interests, current and historical, but acknowledges that some might have been unintentionally missed.

## Supporting information


**Table S1:** fsb271492‐sup‐0001‐Supinfo.docx.

## Data Availability

Data may be made available upon reasonable request to the corresponding author.
