# Supplementary material for: Strenuous Exercise Alters Brain Creatine and Glutamate/Glutamine (Glx) in Humans: Evidence From Dynamic 1H‐MRS and 1H‐MRSI
Source: FASEB J. 2026 Jan 23;40(2):e71492. doi: 10.1096/fj.202504543R (PMC12829524; doi:10.1096/fj.202504543R)
Supplement: Supplementary file 1 — Table S1: fsb271492‐sup‐0001‐Supinfo.docx. [file FSB2-40-e71492-s001.docx]

**Supplementary Material for ‘Strenuous exercise alters brain creatine and glutamate / glutamine (Glx) in humans: evidence from dynamic ^1^H-MRS and ^1^H-MRSI’**

**Table of contents**

**Table S1.** MRSinMRS Checklist.

**Table S2.** Spectral quality metrics for each brain region across each time point.

**Figure S1**. Representative raw spectra from each brain region and reference positioning.

**Figure S2.** Heatmap of the response of total creatine (tCr) and glutamate/glutamine (Glx) to exercise with areas of the primary motor cortex (yellow) and primary somatosensory cortex (green) at the level of the MRSI grid highlighted.

| Table S1. MRSinMRS Checklist | | | |
| --- | --- | --- | --- |
|  | *MRS* | *MRSI* | |
| 1. Hardware |  |  |  |
| a. Field strength [T] | 3 T | 3 T |  |
| b. Manufacturer | Siemens | Siemens |  |
| c. Model (software version if available) | MAGNETOM Vida (XA6) | MAGNETOM Vida (XA6) |  |
| d. RF coils: nuclei (transmit/receive), number of channels, type, body part | 32 channel ^1^H head coil | 32 channel ^1^H head coil |  |
| e. Additional hardware | N/A | N/A |  |
| 2. Acquisition |  |  |  |
| a. Pulse sequence | PRESS | cLASER |  |
| b. Volume of interest (VOI) locations | Prefrontal and visual cortices | Axial slab placed above corpus callosum |  |
| c. Nominal VOI size [cm^3^, mm^3^] | 20 × 20 × 20 mm^3^ | 80 × 80 × 15 mm^3^ |  |
| d. Repetition time (*T* _R_), echo time (*T* _E_) [ms, s] | *T* _R_ = 2000 ms, *T* _E_ = 30 ms | *T* _R_ = 1700 ms, *T* _E_ = 40 ms |  |
| e. Total number of excitations or acquisitions per spectrum  In time series for kinetic studies  i. Number of averaged spectra (NA) per time point  ii. Averaging method (eg block‐wise or moving average)  iii. Total number of spectra (acquired/in time series) | 130 averages | 3 averages |  |
| f. Additional sequence parameters (bandwidth in Hz or dwell time in ms, number of spectral points, frequency offsets). If STEAM: mixing time (*T* _M_). If MRSI: 2D or 3D, FOV in all directions, matrix size, acceleration factors, sampling method | 1200 Hz, 1024 points | 2D: 160 × 160 × 15 mm^3^ FOV; matrix size 16 × 16 |  |
| g. Water suppression method | CHESS | CHESS |  |
| h. Shimming method, reference peak, and thresholds for “acceptance of shim” chosen | Automated 3D *B* _0_ field mapping technique | Automated 3D *B* _0_ field mapping technique |  |
| i. Triggering or motion correction method (respiratory, peripheral, cardiac triggering, incl. device used and delays) | N/A | N/A |  |
| 3. Data analysis methods and outputs | | | |
| a. Analysis software | Osprey (v.2.6.0) | Tarquin (v.4.3.10) | |
| b. Processing steps deviating from quoted reference or product | N/A | N/A | |
| c. Output measure (eg absolute concentration, institutional units, ratio), processing steps deviating from quoted reference or product | Absolute concentration of tCr (mmol·L^-1^) | Signal intensity of tCr (a.u.) | |
| d. Quantification references and assumptions, fitting model assumptions | Default basis set including alanine, aspartate, creatine, gamma-aminobutyric acid, glutamine, glutamate, glycerophosphochlorine, guanidinoacetate, myo-inositol, lactate, N-acetylaspartate, N-acetylaspartylglutamate, phosphorylcholine, phosporylcreatine, scyllo-inositol and taurine. | Default basis set including alanine, aspartate, creatine, gamma-aminobutyric acid, glutamine, glutamate, glycerophosphochlorine, guanidinoacetate, myo-inositol, lactate, N-acetylaspartate, N-acetylaspartylglutamate, phosphorylcholine,phosphorylcreatine, scyllo-inositol and taurine. | |
| 4. Data quality |  |  | |
| a. Reported variables (SNR, linewidth (with reference peaks)) | SNR, linewidths, and CRLBs | SNR, linewidths, and CRLBs | |
| b. Data exclusion criteria | CRLB <20% (<40% allowed for lactate), SNR >10 and FWHM <15 Hz. | CRLB <20% (<40% allowed for lactate), SNR >10 and FWHM <15 Hz. | |
| c. Quality measures of postprocessing model fitting (eg CRLB, goodness of fit, SD of residual) | Table S2 | Table S2 | |
| d. Sample spectrum | Figure S1 | Figure S1 | |

| Table S2. Spectral quality metrics for each brain region across each time point | | | | | | | | |
| --- | --- | --- | --- | --- | --- | --- | --- | --- |
|  | Frontal cortex | | | Visual cortex | | | MRSI grid | |
|  | *PRE* | *POST 15* | *POST 30* | *PRE* | *POST 15* | *POST 30* | *PRE* | *POST 25* |
| SNR | 74.7 (12.8) | 71.9 (13.8) | 72.0 (15.0) | 78.5 (24.9) | 78.4 (22.3) | 82.8 (23.4) | 27.2 (6.6) | 29.1 (6.0) |
| FWHM (Hz) | 8.7 (1.7) | 8.4 (1.5) | 9.0 (1.7) | 7.2 (0.5) | 7.2 (0.6) | 7.4 (0.6) | 9.3 (2.3) | 9.5 (2.1) |
| CRLB tCr (%) | 2.6 (0.7) | 2.6 (1.0) | 2.8 (0.9) | 2.0 (0.2) | 2.0 (0.3) | 2.1 (0.5) | 2.3 (1.0) | 2.6 (1.1) |
| CRLB Glx (%) | 6.2 (1.0) | 6.1 (1.6) | 6.3 (1.5) | 3.8 (0.8) | 3.8 (0.4) | 3.7 (0.6) | 6.9 (2.4) | 6.0 (2.3) |
| SNR: signal to noise ratio; FWHM: full width at half maximum; CRLB: Cramér–Rao Lower Bound; tCr: total creatine; Glx: glutamate and glutamine. | | | | | | | | |


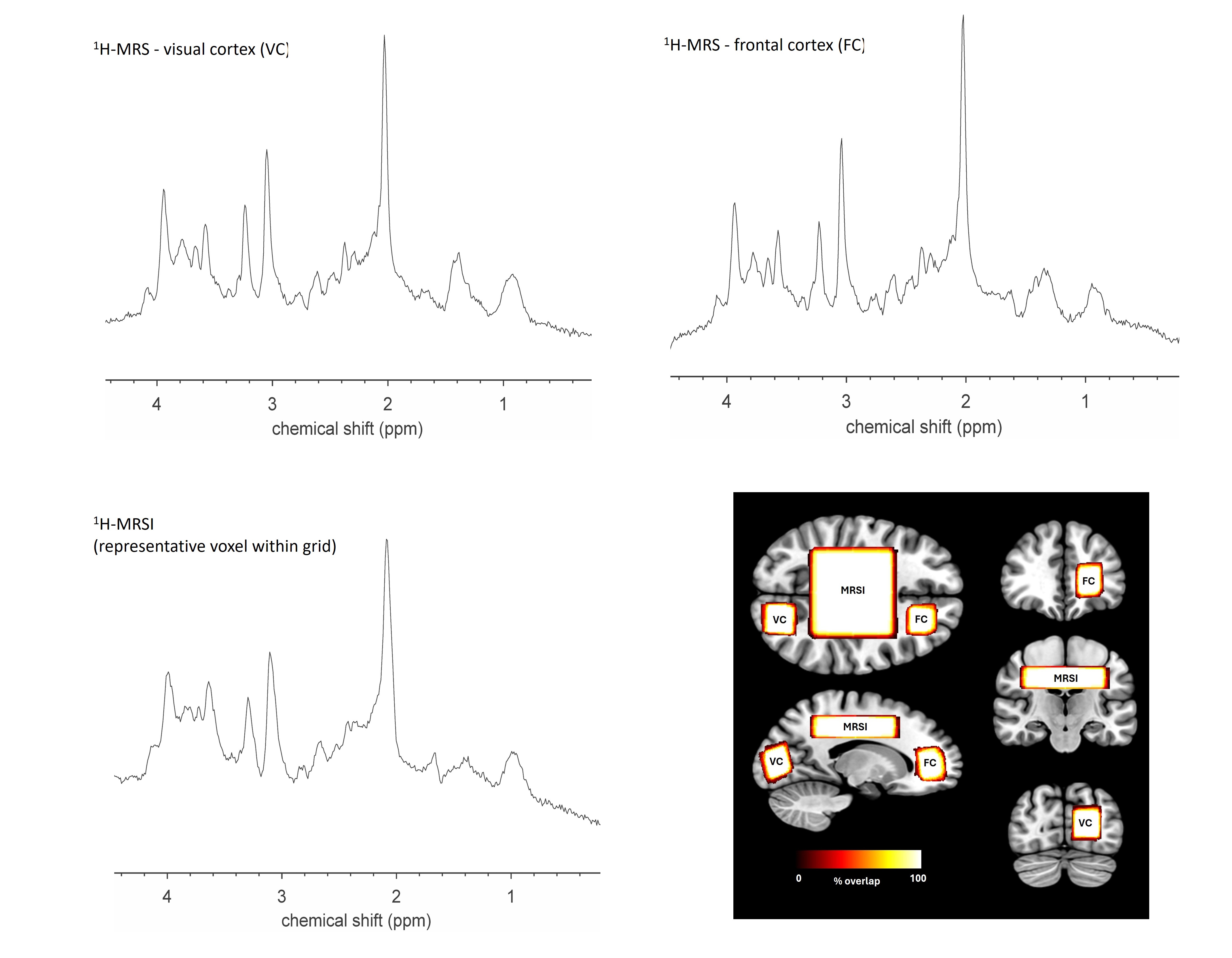


**Figure S1**. Representative raw spectra from each brain region and reference positioning


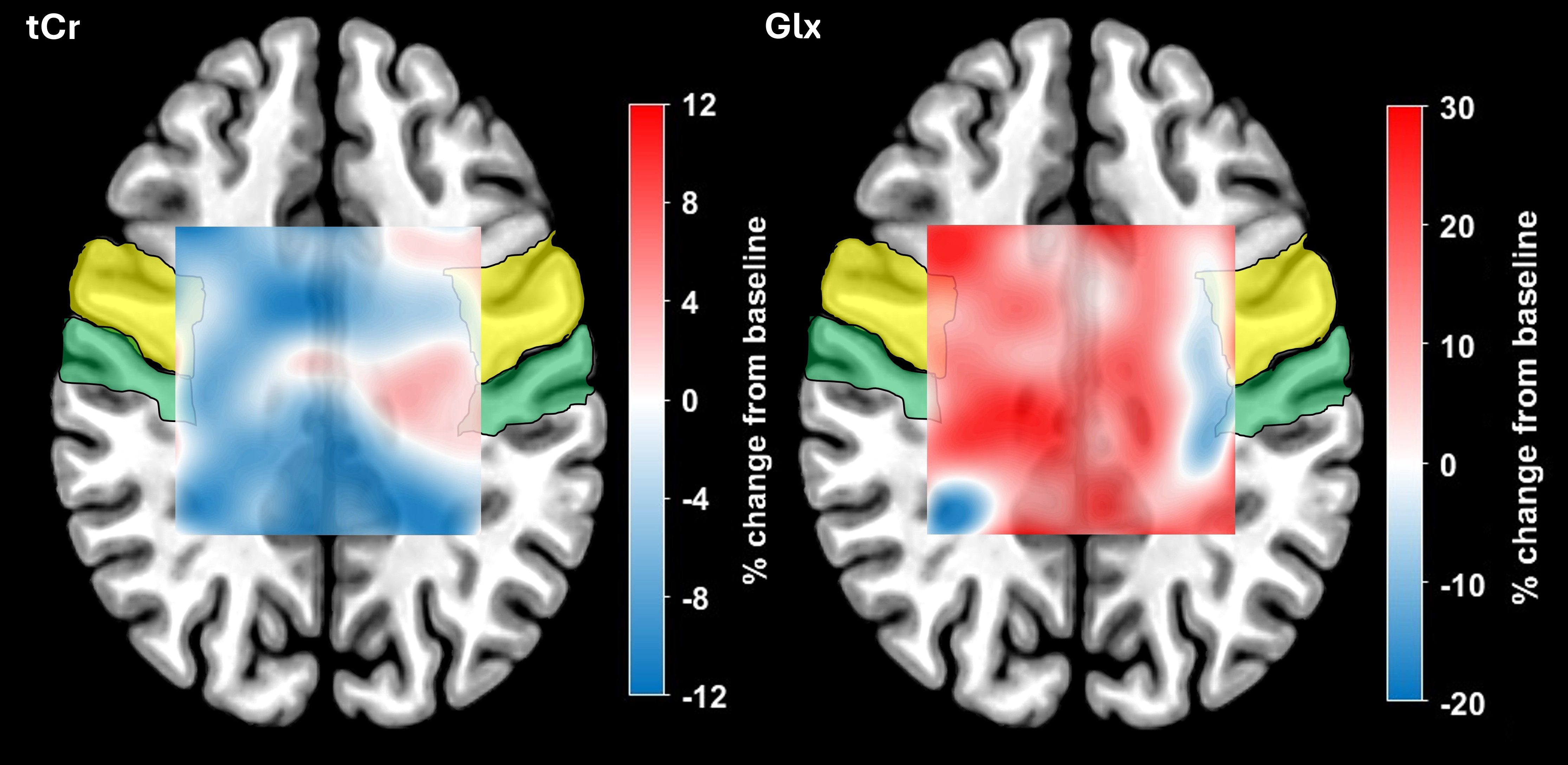


**Figure S2.** Heatmap of the response of total creatine (tCr) and glutamate/glutamine (Glx) to exercise with areas of the primary motor cortex (yellow) and primary somatosensory cortex (green) at the level of the MRSI grid highlighted
